# Exome sequencing identifies novel variants associated with non-syndromic hearing loss in the Iranian population

**DOI:** 10.1371/journal.pone.0289247

**Published:** 2023-08-10

**Authors:** Jalal Vallian Broojeni, Arezu Kazemi, Halimeh Rezaei, Sadeq Vallian

**Affiliations:** Department of Cell and Molecular Biology& Microbiology, Faculty of Science and Technology, University of Isfahan, Isfahan, IR, Iran; Kingston University London / Hamad Bin Khalifa University (HBKU) / Women’s Wellness and Research Center (WWRC), QATAR

## Abstract

Autosomal recessive non-syndromic hearing loss (ARNSHL) is a public health concern in the Iranian population, with an incidence of 1 in 166 live births. In the present study, the whole exome sequencing (WES) method was applied to identify the mutation spectrum of NSHL patients negative for *GJB2* gene mutations. First, using ARMS PCR followed by Sanger sequencing of the *GJB2* gene, 63.15% of mutations in patients with NSHL were identified. Among the identified mutations in *GJB2*:p.Val43Met and p.Gly21Arg were novel. The remaining patients were subjected to WES, which identified novel mutations including *MYO15A*:p.Gly39LeufsTer188, *ADGRV1*:p.Ser5918ValfsTer23, *MYO7A*: c.5856+2T>c (splicing mutation), *FGF3*:p.Ser156Cys. The present study emphasized the application of WES as an effective method for molecular diagnosis of NSHL patients negative for *GJB2* gene mutations in the Iranian population.

## Introduction

Hearing loss (HL) is considered one of the most frequent sensory impairments in humans, affecting all age groups and genders with an incidence of 1–2 in 1000 neonates in the world [1]. It has been documented that approximately 50–70% of cases of HL are related to genetic causes [2]. Hereditary HL can be regarded as syndromic or non-syndromic. Non-syndromic hearing loss is a partial or total loss of hearing with no other signs and symptoms. In contrast, syndromic hearing loss occurs with signs and symptoms affecting other parts of the body. According to severity, HL is classified into four grades (mild, moderate, severe, and profound) [3]. Non-syndromic hearing loss (NSHL) shows different modes of inheritance, including autosomal recessive (AR), autosomal dominant (AD), X‐linked, and mitochondrial [4]. AR transmission accounts for 75%–85% of all cases, while AD inheritance accounts for 15%–25% of cases. A small proportion of cases (1%–2%) show X‐linked or mitochondrial inheritance [5].

To date, over 60 mapped loci have been identified for NSHL as a highly heterogeneous condition. The reports indicate that mutations in one locus, *DFNB1* (13q11-12) which contains *GJB2* (NM_004004.5) and *GJB6* (NM_001110219.2) genes, make up 50% of the etiology in many populations [6]. *GJB2* encodes the connexin 26 protein (Cx26), which is a member of the connexin protein family, forms channels called gap junctions, and is involved in inner ear homeostasis through the recycling of potassium ions. The *GJB6* gene structure is thought to be relatively simple, consisting of only two exons, with one untranslated exon (exon 1) [7]. Overexpression of *GJB2* has been found to be associated with a poor prognosis in several human cancers [8–10]. It has been reported that GJB2 expression was negatively associated with progression status in breast cancer tissues and may function as a regulator of breast tumorigenesis. Moreover, the knockdown of *GJB2* in human breast cancer cell lines using shRNA resulted in a significant decrease in the proliferative ability and an increase in the migratory ability of breast cancer cells [11]. Until now, more than 100 pathogenic variants in the *GJB2* gene have been reported to cause ARNSHL [12]. The prevalence of *GJB2* mutations is different between populations. For instance, in Caucasians, c.35delG is the most frequent mutation leading to deafness. Distribution of the c.35delG frequency in carriers in this population is as high as 2–4% [13]. However, c.167delT in the Ashkenazi Jewish [14], c.235delC in the Japanese [15] and p.Trp24* in the Indians [16] are the most common mutations.

Several studies have shown that the *GJB2* mutations *have contributed* to *16–18*% of *ARNSHL* in the Iranian population *[17–19]*. Moreover, the frequency of the mutations differs from 86.7% in Finland [20] to 57.5%, 33.3%, 25%, 3.7% and 0% in Lithuania [21], Croatia [22], Turkey [23], Pakistan [24] and Oman [25], respectively. However, in this population, the frequency of *GJB2* mutation varies in different regions. In the South part of Iran, it is less than 4% but increases to 27.5% in the North and Northwest of Iran [26]. Moreover, studies on a large number of families with ARNSHL from Sistan and Baluchistan province in the Southeast of Iran indicated that 7% of the population had mutations in the *GJB2* gene. Interestingly, the *GJB2*:c.35delG mutation which is the most frequent mutation in different regions of Iran was not observed in the Sistan and Baluchistan province and the most prevalent mutation was p.Trp24*(80% from all *GJB2* reported mutations) [27]. These findings were comparable to the presented data from a Pakistani population that is geographically close to Sistan and Baluchistan province of Iran [24]. Additionally, about 26% of Iranian Azeri (northwest of Iran) ARNSHL patients’ etiology is due to mutations in the *GJB2* gene [28]. In this population, it has been stated that c.35delG accounted for about 62% of the *GJB2* mutations. Moreover, the results of a study on a Turkish population which is close to the Iranian Azeri population, were in line with this study [23]. Together, these reports showed the presence of an unequal distribution in the prevalence of *GJB2*-related ARNSHL throughout the Iranian population.

However, there is no defined strategy for the diagnosis of NSHL disease-causing mutations in this population. In the present study, using ARMS-PCR followed by Sanger and whole exome sequencing, it was possible to detect almost the entire disease-causing mutations among the patients affected with NSHL in the Iranian population.

## Materials and methods

### Sample collection

A total of 76 patients affected with non-syndromic hearing loss (NSHL) mostly from consanguineous marriage (90.79%) which were referred to Isfahan medical genetics center (www.isfahangenetics.com) were included in this study. All the patients were diagnosed with NSHL and referred by the audiologist. The clinical history of each proband was explored to ensure that the hearing loss was not a result of infection, trauma, acoustic trauma, or ototoxic drugs. The patients had no other clinical manifestation except hearing loss.

In this study, written consent was obtained from all participants and parental consent was obtained when the patient was a child. All procedures were approved by University of Isfahan institutional review board (IRB) for research and ethics approval (Approval Ref No. 790205). A total of 122 individuals was tested including 76 probands and 46 probands’ family members who participated in the co-segregation analysis.

Blood samples were collected at Isfahan medical genetics center. Genomic DNA was extracted from peripheral blood mononuclear cells of patients and their family members by salting out procedure [29]. The DNA was quantified and stored at room temperature for daily experiments and kept at -20°C for future applications.

### Genotyping

Mutation analysis was performed first for the detection of *GJB2*: c.35delG mutation using amplification refractory mutation analysis system PCR (ARMS-PCR) [30]. Two PCR assays using the normal or mutant primer along with the common and control primer were used (Table 1). Then, Sanger sequencing of exon 2 of *GJB2* was performed for samples that were negative for any pathogenic or likely pathogenic variants in ARMS-PCR. The primer used for ARMS-PCR and sequencing (Table 1) were designed using Primer3 (bioinfo.ut.ee/primer3-0.4.0/). PCR reactions for sequencing were performed under the following conditions: initial denaturation at 95°C / 4 minutes, followed by 30 cycles of denaturation at 95°C/30 seconds, annealing at 59°C/30 seconds, elongation at 72°C/45 seconds, and extension at 72°C/5 minutes.

**Table 1:** Primers for *GJB2*:c.35DelG screening (ARMS PCR).

	Primer name	Sequence (5’ to 3’)
**ARMS-PCR (GJB2:c.35DelG screening)**	35DelGN (Normal Allele)	TTGGGGCACGCTGCAGACGATCCTGGGGAG
	35DelGM (Mutant Allele)	TTGGGGCACGCTGCAGACGATCCTGGGGAT
	35DelGC (common)	GAAGTAGTGATCGTAGCACACGTTCTTGCA
	AAT3 (Control)	CCCACCTTCCCCTCTCTCCAGGCAAATGGG
	AAT4 (Control)	GGGCCTCAGTCCCAACATGGCTAAGAGGTG
**Sanger Sequencing**	GJB2E2F	CTCCCTGTTCTGTCCTAGCT
	GJB2E2R	CTCATCCCTCTCATGCTGTC

### Whole exome sequencing

For whole exome sequencing (WES), the library preparation was performed using the SureSelectTX kit (Agilent, USA) following the instruction recommended by the provider. Sequencing was carried out using NovaSeq 6000 Sequencing System (Macrogen Co., South Korea). Sequence reads were aligned against the human reference genome (hg19, NCBI Build 37) using Burrows-Wheeler Aligner (https://bio-bwa.sourceforge.net/). SAMtools v1.0 (http://github.com/samtools/samtools) was used to identify quality-filtered single nucleotide substitutions and small insertion deletions (filtered variants with a quality score ≥20). Variant annotation was done by the wANNOVAR online software tool (http://wannovar.wglab.org/).

According to the American College of Medical Genetics and Genomics (ACMG) guidelines, different criteria like frequency of variations in the population database, mode of inheritance, and prediction software were used to filter out detected variants [31]. Minor allele frequency with a cutoff value of <0.05 in population databases was used for filtering variants. Moreover, the pathogenicity predicting software tools including SIFT [32], PolyPhen2.0 [33], MutationTaster2 [34], PROVEAN [35], FATHMM [36], and CADD [37] were used. Based on the results obtained from these methods, the effect of the variant of interest on protein function was predicted.

### Variant validation and co-segregation analysis

Validation of variant calls and phenotype of all available index cases and family members of the affected individuals were carried out using Sanger sequencing. (ABI-3500, Thermo Fisher Scientific, USA). The sequencing results were analyzed by BioEdit (version 7) [38].

## Results and discussion

This study was performed on 76 patients diagnosed with NSHL from the central provinces of Iran (Patients’ demographic data mentioned at S1 Table). In the first step, all the patients were screened for *GJB2*:c.35delG mutation using the ARMS-PCR method. The results showed that 35 patients (46.05%) were homozygous for this mutation. The patients who were homozygous for 35delG were affected by profound hearing loss (consisting of 10 males and 25 females). Interestingly, except for one family, most positive cases for 35delG were from consanguineous marriages. Next, to find other mutations in *GJB2*, exon 2 of the *GJB2* gene (the only coding exon of the gene) was fully sequenced in the remaining 41 negative cases. Among the patients examined, six patients had homozygous variants, and seven had only one heterozygous mutation for *GJB2*. As mentioned in Table 2, all variants identified in exon 2 of *GJB2* were classified pathogenic or likely pathogenic based on ACMG guideline. However, for 28 patients no mutation was detected. All the mutations identified using Sanger sequencing were presented in Table 2.

**Table 2:** Pathogenic and likely pathogenic variants identified by Sanger sequencing of *GJB2*:Exon2.

#	Mutation	rs number	Zygosity	ACMG	ClinVar	Novel variant/Reference	Phenotype	Gender
**1**	*GJB2*(NM_004004.6):c.167del: (p.Leu56ArgfsTer26)	rs80338942	Homo	Likely Pathogenic	Pathogenic	No/ ClinVar ID: 17010	profound SNHR	Male
**2**	*GJB2*(NM_004004.6):c.358_360del: (p.Glu120del)	rs80338947	homo	Pathogenic	Pathogenic	No/ ClinVar ID: 17006	profound SNHR	Female
**3**							profound SNHR	Male
**4**	*GJB2*(NM_004004.6):c.299_300del: (p.His100ArgfsTer14)	rs111033204	homo	Pathogenic	Pathogenic	No/ ClinVar ID: 44736	profound SNHR	Female
**5**							profound SNHR	Female
**6**	*GJB2*(NM_004004.6):c.427C>T: (p.Arg143Trp)	rs80338948	homo	Pathogenic	Pathogenic	No/ ClinVar ID: 17009	profound SNHR	Female
**7**	*GJB2*(NM_004004.6):c.224G>A: (p.Arg75Gln)	rs28931593	Het	Pathogenic	Pathogenic	No/ ClinVar ID: 17027	profound SNHR	Male
**8**	*GJB2*(NM_004004.6):c.23C>T: (p.Thr8Met)	rs529500747	Het	Likely Pathogenic	Conflicting interpretations of pathogenicity​ Likely pathogenic(5); Uncertain significance(3)	No/ ClinVar ID: 379889	profound SNHR	Female
**9**	*GJB2*(NM_004004.6):c.127G>A: (p.Val43Met)	-	Het	Likely Pathogenic	-	YES	profound SNHR	Male
**10**	*GJB2*(NM_004004.6):c.292C>T: (p.Arg98Trp)	rs529440698	Het	Likely Pathogenic	-	NO/ [39]	profound SNHR	Female
**11**	*GJB2*(NM_004004.6):c.487A>G: (p.Met163Val)	rs80338949	Het	Pathogenic	Conflicting interpretations of pathogenicity​ Likely pathogenic(1);Uncertain significance(4)	No/ ClinVar ID: 21388	profound SNHR	Female
**12**	*GJB2*(NM_004004.6):c.61G>A: (p.Gly21Arg)	-	Het	Likely Pathogenic	-	Yes	profound SNHR	Female
**13**	*GJB2*(NM_004004.6):c.230G>A: (p.Trp77Ter)	rs104894395	Het	Pathogenic	Pathogenic	No/ ClinVar ID: 189176	profound SNHR	Male

Homo: Homozygote, het: Heterozygote, SNHL: Sensorineural hearing loss

The results from screening using Sanger sequencing of *GJB2*: exon2 showed that 13 of 76 cases (17.10%), were positive. Among these positive cases, 6 cases were homozygous and 7 were heterozygous. It is important to note that all the cases except 28867 and 222597 were from the results of consanguineous parents. Interestingly, in patient 28867, the identified mutation (rs28931593) is a pathogenic variant that was reported before and causes an autosomal dominant phenotype. However, in case 222597, the identified mutation (rs529500747) is a variant that its association with NSHL is controversial [40–42] (Table 3). This indicates that it is likely that there be a second mutation (variation) in other related genes such as *GJB3* or *GJB6* that may be the cause of the deafness in a compound heterozygous situation.

**Table 3:** Statistics of identified variants by different methods.

#	Positive for *GJB2*:c.35DelG	Positive for sanger sequencing of *GJB2* exon 2	A positive result from the WES test	A negative result from m WES test	Total cases
**Number of case**	35	13	24	4	76
**Percentage of case**	46.05	17.10	31.58	5.26	100

To identify disease-causing mutations in the remaining negative cases, whole exome sequencing (WES) was carried out. Among the patients analyzed by WES, one pathogenic or likely pathogenic variant related to hearing loss was identified for 85.7% of the patients. However, 4 patients (14.28%) were negative for any point mutation and/or small deletions. These results were presented in Table 4. Allele frequency, scores of pathogenicity prediction software related to identified variants by WES mentioned at S2 Table.

**Table 4:** The spectrum of variants identified by Whole Exome Sequencing (WES) in patients with NSHL in the Iranian population.

#	consanguinity	Gene	Mutation	rs Number	Heterozygosity/ Inheritance	ACMG	ClinVar	Novel variant/ Reference	Phenotype	Gender
**1**	Yes	*MYO15A*	*MYO15A*:NM_016239.4:c.10572dup:p.Ser3525GlnfsTer79	rs1057519607	Homo/AR	Pathogenic	Pathogenic	No/ ClinVar ID: 375679 [43]	Severe-profound SNHL	Female
**2**	Yes		*MYO15A*(NM_016239.4):c.4108C>T (p.Arg1370Cys)	rs878854411	Homo/AR	Likely Pathogenic	Uncertain significance	No/ ClinVar ID: 242330	Severe-profound SNHL	Female
**3**	Yes		*MYO15A*(NM_016239.4):c.7720C>T (p.Gln2574Ter)	rs757066608	Homo/AR	Pathogenic	Pathogenic	No/ [44]	Severe-profound SNHL	Female
**4**	Yes		*MYO15A*(NM_016239.4):c.114_115del and MYO15A:c.118_119del, (p.Gly39LeufsTer188)	-	Homo/AR	Pathogenic	-	Yes	Severe-profound SNHL	Female
**5**	Yes	*OTOF*	*OTOF*(NM_194248.3):c.1966del (p.Arg656GlyfsTer10)	rs397515590	Homo/AR	Pathogenic	Pathogenic	No/ ClinVar ID: 65786	Severe-profound SNHL	Male
**6**	Yes		*OTOF*(NM_194323.3):c.3515G>A (p.Arg1172Gln)	rs201326023	Homo/AR	Pathogenic	Pathogenic	No/ ClinVar ID: 548986	Severe-profound SNHL	Male
**7**	Yes		*OTOF*(NM_194248.3):c.1981dup (p.Asp661GlyfsTer2)	-	Homo/AR	Pathogenic	-	No/ [45]	Severe-profound SNHL	Male
**8**	NO	*MYO7A*	*MYO7A*(NM_000260.4):c.3659C>T (p.Pro1220Leu)	rs727504710	Het/AD	Likely Pathogenic	Uncertain significance​	No/ ClinVar ID: 179208	Moderate, SNHL	Male
**9**	Yes		*MYO7A*:c.5856+2T>c	-	Homo/AR	Pathogenic	-	Yes	Severe NHL	Male
**10**	Yes		*MYO7A*(NM_000260.4):c.73G>A (p.Gly25Arg)	rs782252317	Homo/AR	Pathogenic	Pathogenic	No/ ClinVar ID: 177722	Profound SNHL, retinitis pigmentosa	Male
**11**	Yes	*OTOA*	*OTOA*(NM_144672.4):c.635+2T>A	rs745871771	Homo/AR	Pathogenic	-	No/ ExAC ID: NC_000016.9–21698971	Moderately SNHL	Female
**12**	Yes	*TRIOBP*	*TRIOBP*(NM_001039141.3):c.3073C>T (p.Arg1025Ter)	rs776962899	Homo/AR	Pathogenic	Pathogenic	No/ ClinVar ID: 930753	Moderately SNHL	Female
**13**	Yes	*SLC26A4*	*SLC26A4*(NM_000441.2):c.1489G>A (p.Gly497Ser)	rs111033308	Homo/AR	Pathogenic	Pathogenic	No/ ClinVar ID: 43510	Severe SNHL	Male
**14**	Yes	*CDC14A*	*CDC14A*(NM_003672.4):c.1126C>T (p.Arg376Ter)	rs876661408	Homo/AR	Pathogenic	Pathogenic	No/ ClinVar ID: 235145	Severe SNHL	Female
**15**	Yes	*PDZD7*	*PDZD7*(NM_001195263.2):c.166dup (p.Arg56ProfsTer24)	rs587776894	Homo/AR	Pathogenic	Pathogenic	No/ ClinVar ID: 30983	Moderately SNHL	Male
**16**	No	*WFS1*	*WFS1*(NM_006005.3):c.2590G>A (p.Glu864Lys)	rs74315205	Het/AD	Pathogenic	Pathogenic	No/ ClinVar ID: 4526	Moderate SNHL	Male
**17**	Yes	*SMPX*	*SMPX*(NM_014332.3):c.99del (p.Arg34GlufsTer47)	rs398122930	Hemi/XLD	Pathogenic	Pathogenic	No/ ClinVar ID: 40063 [46]	Moderately severe SNHL	Male
**18**	Yes	*GJB2*	*GJB2*:UTR5:(NM_004004.6):c.-23+1G>A	rs80338940	Homo/AR	Pathogenic	Pathogenic	No/ ClinVar ID: 17029	Profound SNHL	Female
**19**	No	*MYH14*	*MYH14*(NM_001145809.2):c.359C>T (p.Ser120Leu)	rs119103281	Het/AD	Pathogenic	Pathogenic	No/ ClinVar ID: 2200	Severe-profound SNHL	Male
**20**	Yes	*TSPEAR*	*TSPEAR*(NM_144991.3):c.1728del:(p.Lys577SerfsTer37)	rs782540538	Homo/AR	Pathogenic	Pathogenic	No/ ClinVar ID: 37311	Profound SNHL	Male
**21**	Yes	*FGF3*	*FGF3*(NM_005247.4):c.467C>G (p.Ser156Cys)	-	Homo/AR	Likely Pathogenic	-	Yes	Profound SNHL, microtia	Female
**22**	Yes	*ADGRV1*	*ADGRV1*(NM_032119.4):c.17752_17755del (p.Ser5918ValfsTer23)	-	Homo/AR	Pathogenic	-	Yes	Moderate SNHL	Female
**23**	Yes	*COL11A2*	*COL11A2*: NM_080680:exon8:c.966dupC:p.Thr323HisfsTer19	rs748440351	Homo/AR	Likely Pathogenic	Conflicting interpretations of pathogenicity​ Pathogenic(5); Likely pathogenic(2); Uncertain significance(2)	No/ ClinVar ID: 497724	Moderate SNHL	Male
**24**	Yes								Moderate SNHL	Male

Homo: Homozygote, het: Heterozygote, SNHL: Sensorineural hearing loss

**Fig 1:**
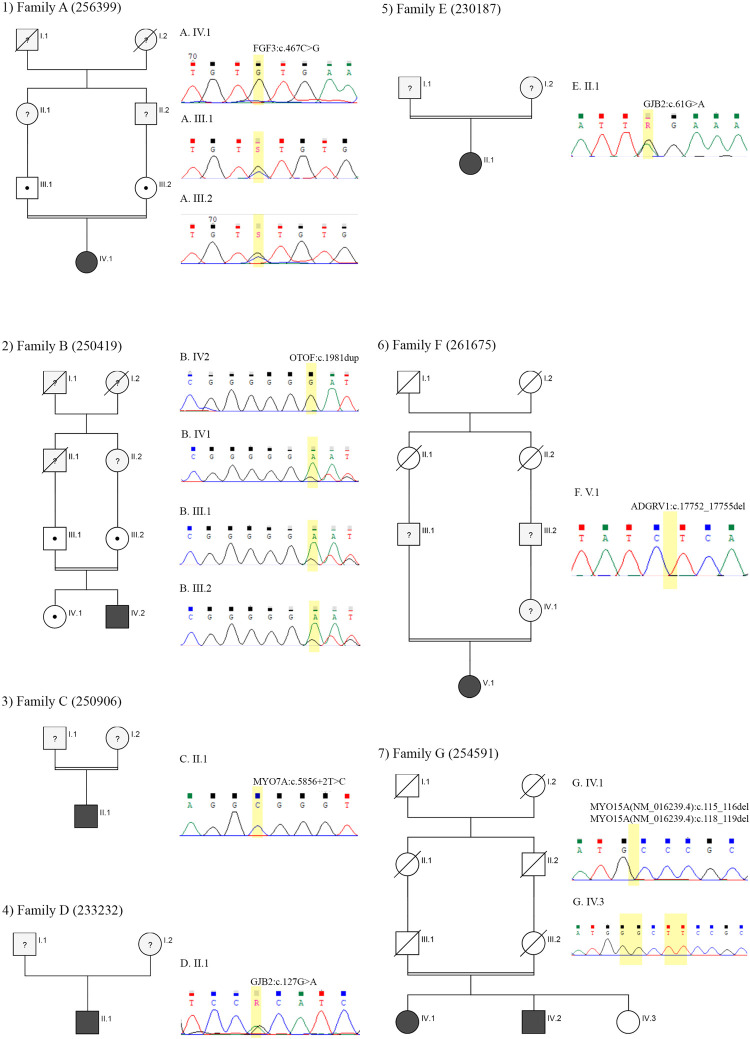
Pedigrees and sequence chromatograms of novel identified variants. Representation of pedigrees and chromatograms of novel variants in seven unrelated families with non-syndromic hearing loss were shown. Abbreviations and symbols are as follows: The arrow represents the proband, black represents deafness, the cross represents death, the question mark represents the unknown and the dot represents carrier. For details regarding each variant, see Table 3.

Of these mutations which were identified by WES, four mutations were not previously reported and could be considered novel (Fig 1). Segregation analysis of novel variants in the proband’s available family members is mentioned in Fig 1. These mutations consisted of i) *MYO15A*:c.115_116del and *MYO15A*:c.118_119del; and ii) *ADGRV1*:c.17752_17755del which were both deletion mutations with stop gain codon, iii) *MYO7A*:c.5856+2T>c which could result in the disruption of splicing and iv) *FGF3*:c.467C>G which is a missense mutation. An alternative variant at this position of *FGF3* (chr11:69625327 A⇒G (Ser156Pro)) was previously reported and classified as pathogenic by ClinVar [47]. *OTOF*:c.1981dup is a duplication variant that causes a stop gain codon. It has been previously reported in an Iranian family, but yet has not been included in any databases [45].

The clinical significance of the variants identified by WES was included in Table 4. As presented, of 24 cases that were positive by WES, 19 patients had pathogenic variants (79.16%), 3 cases (12.5%) were likely pathogenic and 2 cases (8.3%) had a variation with uncertain significance classification (see Table 5).

**Table 5:** ACMG classification of variant identified by WES.

ACMG classification	Pathogenic	Likely Pathogenic	Total cases negative for GJB2 mutation
**#cases**	19	5	24
**percentage of cases %**	79	21	100

It is noticeable that one pathogenic variant *GJB2*:NM_004004.6):c.-23+1G>A in UTR of *GJB2* was identified by WES. This region is not usually included in Sanger sequencing methods, suggesting the importance of UTRs of the *GJB2* gene in the identification of the cause of the ARNSHL disease.

Mutations in myosin-VIIa (MYO7A) cause three types of diseases: I) Deafness, autosomal dominant 11 (601317), II) Deafness, autosomal recessive 2 (600060) III) Usher syndrome, type 1B (276900). In this study, all three types have been observed: Proband 263097 is a male with an onset of mild hearing loss at 4 years old, now he is 13 years old. WES identified a heterozygous substitution (Pro1220Leu) in MYO7A that was reported as a variant of uncertain significance in ClinVar in association with hearing loss but based on ACMG guideline this variant was classified as likely pathogenic. Proband 261505 is 10 years old boy with an onset of profound sensorineural hearing at birth and retinitis pigmentosa at 6 years old. A pathogenic homozygous variant (Gly25Arg) at MYO7A was identified for him. Proband 250906 is 55 years old male with profound hearing loss from birth. WES identified a novel variant in MYO7A (c. 5856+2 T>C) that may disrupt the splicing of Exon 42 which has a severe effect on protein domains.

The majority of MYO15A variants are associated with a congenital severe to profound non-syndromic hearing loss phenotype except for some exon 2 variants [48]. In this study, four variants in MYO15A have been identified: three mutations are termination mutation (exon 2, 4,0, and 66) and one substitution (exon 9). All four homozygous mutations identified in this study are classified as pathogenic or likely pathogenic based on ACMG guideline and all patients are affected by congenital severe to profound non-syndromic hearing loss (Table 4). A variant was found in exon 2 of the *MYO15A* gene as two executive small deletions as MYO15A(NM_016239.4):c.114_115del and MYO15A:c.118_119del, (p.Gly39LeufsTer188) is a novel mutation that causes termination of the MYO15A protein. The presence of this mutation/deletion shows severe effects on the predicted protein structure. Patients carrying this mutation in a homozygous state display a severe form of hearing loss.

*OTOF*-related deafness is characterized by two phenotypes: prelingual nonsyndromic auditory neuropathy spectrum disorder (ANSD) and, less frequently, temperature-sensitive auditory neuropathy spectrum disorder (TS-ANSD). Three pathogenic variants were identified in *OTOF* consisting of one deletion, one duplication, and one substitution. c.1966del and c.3515G>A are pathogenic variants that were reported previously, but c.1981dup is a novel variant that causes termination of the protein. All three patients are affected with congenital severe and profound SNHL.

**Fig 2:**
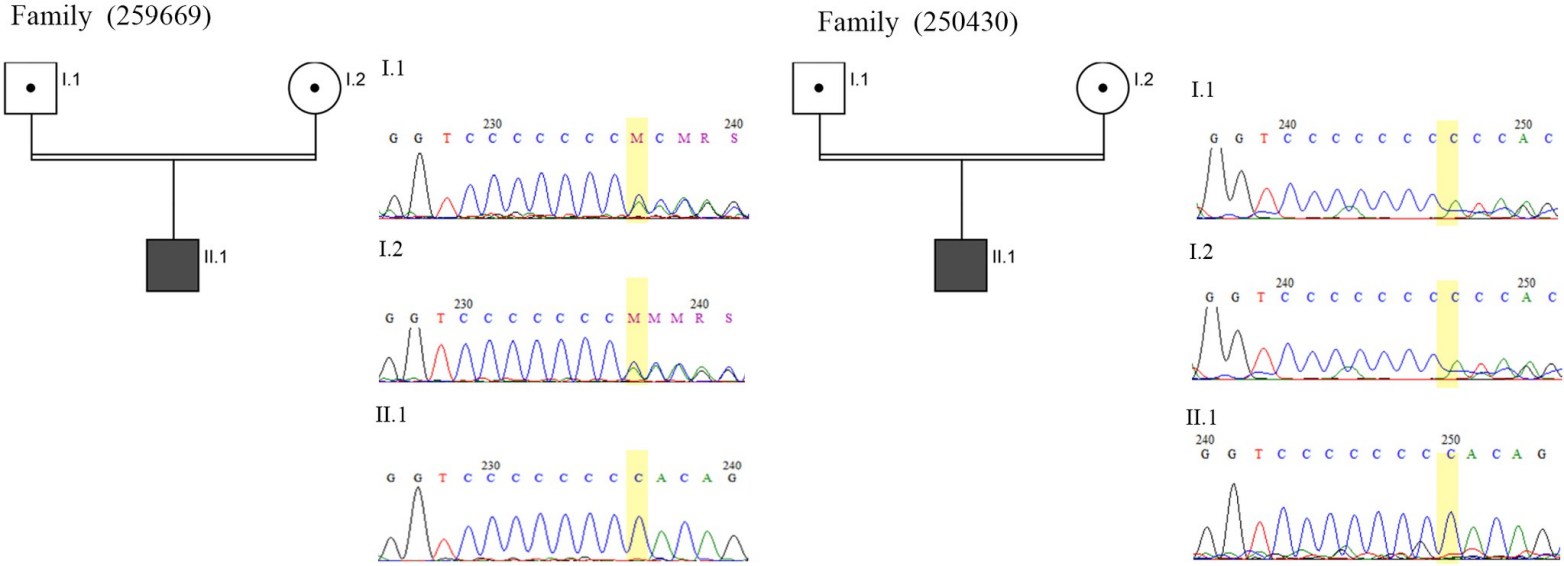
Pedigrees and sequence chromatograms of *COL11A2*:c.966dupC in two different families.

Moreover, among the variations identified *COL11A2*:NM_080680:exon8:c.966dupC:p.Thr323HisfsTer19 is a variant with conflicting interpretations of pathogenicity, which was identified in two patients from two different families with consanguineous marriages (Table 2). Interestingly, this variant was reported as a cause of hearing loss in Ellis-van Creveld Syndrome with hearing loss in an Iranian child. In this patient with two distinct phenotypes, a mutation in *EVC2* (c.2653C>T; p.Arg885*) was the reason for Ellis-van Creveld Syndrome and *COL11A2*:c.966dupC for hearing loss [49]. Therefore, this study along with two more cases and co-segregation in their families can support the pathogenicity of this variant (Fig 2).

Previous studies have shown the association of mutations in *GJB2*, *SLC26A4*, and *TECTA* genes with high priority and in genes *MYO15A*, *ILDR1*, *TMC1*, *PJVK*, *LRTOMT*, *MYO7A*, *OTOF*, and *MARVELD2* with less significance with ARNSHL in the Iranian population [26]. The data from the present study indicated that GJB2 (c.35delG: 46.05% of cases), *MYO15A* (4 families), *OTOF* (3 families) and *MYO7A* (3 families) could be considered as more distinct pathogenic variants in the Iranian population (Table 2). Mutations in *GJB2*, especially *GJB2*:c.35delG, were of most importance in the central regions of Iran and its frequency is in line with north of Iran but not Southeast of Iran. The variants reported in the present study along with other frequent variants will help genetic counseling and family planning for families with deaf patients, which is expected to affect the frequency of deaf individuals in the Iranian population.

All the Iranian patients included in the present study were non-syndromic and had no history of cancer. Moreover, other studies in Iran did not report any cancer associated with *GJB2*-related ARNSHL [50]. This might be explained by the notion that the increased expression of GJB2 has been associated with the progression of different cancers, indicating an oncogenic role for the GJB2 protein [11]. However, in *GJB2*-related ARNSHL patients, usually there is a lack of normal expression (abnormal mRNA and or protein expression) of GJB2 protein. Besides, it is speculated that defects in the *GJB2* gene might function toward the prevention of cancer in *GJB2*-related ARNSHL which needs further investigation.

According to the observation that even non-consanguineous parents were carriers of mutations, it is assumed that the funder effects might be considered one of the important factors in the prevalence of the ARNSHL disease in the Iranian population. Moreover, the study population which is almost from Isfahan province encompasses various sects today. The majority of the people in the province are Persian but Bakhtiari Lurs, Kurds, Georgians, Armenians, Qashqais, and Persian Jews. This may indicate that the population is almost isolated and the inbreeding rate within each ethnicity is high. Therefore, the effects of possible genetic drift could be considered. It seems that among the world’s most heterogeneous populations, Iran has received a great deal of attention as a potential risk factor for different autosomal recessive disorders.

## Conclusion

In the present study, for the first time, using whole exome sequencing (WES), the mutation spectrum of patients with NSHL who were negative for mutations in the *GJB2* gene was depicted and novel mutations were identified in the Iranian population. Despite the high allele frequency of *GJB2*:35delG in the central region of Iran, the data showed that WES could be considered a convenient and cost-effective tool for the identification of the genetic cause of heterogenic diseases like NSHL compare to ARMS-PCR and Sanger sequencing.

## Supporting information

S1 Table Patient demographic. Gender, age and ethnicity of probands were shown.
(DOCX)

S2 Table Whole exome sequencing variants data. Allele frequency in population databases and the pathogenicity of each variant in different pathogenicity prediction software were mentioned.
(XLSX)
