# Supplementary material for: Exome sequencing identifies novel variants associated with non-syndromic hearing loss in the Iranian population
Source: PLoS One. 2023 Aug 10;18(8):e0289247. doi: 10.1371/journal.pone.0289247 (PMC10414579; doi:10.1371/journal.pone.0289247)
Supplement: S1 Table — Gender, age and ethnicity of probands were shown. (DOCX) [file pone.0289247.s001.docx]

| Gender | |
| --- | --- |
| Female | 44 |
| Male | 32 |
| Ethnicity | |
| Persian | 47 |
| Bakhtiari Lur | 18 |
| Qashqai Turk | 11 |
| Range of age (years old) | |
| Less than 10 | 4 |
| 11-20 | 17 |
| 21-30 | 11 |
| 31-40 | 26 |
| More than 40 | 18 |
| Geographic region (province) | |
| Esfahan | 62 |
| Chaharmahal and Bakhtiari | 12 |
| Lorestan | 2 |
